# ILDR2 stabilization is regulated by its interaction with GRP78

**DOI:** 10.1038/s41598-021-87884-7

**Published:** 2021-04-16

**Authors:** Kazuhisa Watanabe, Kazuhiro Nakayama, Satoshi Ohta, Ayumi Matsumoto, Hidetoshi Tsuda, Sadahiko Iwamoto

**Affiliations:** 1 Division of Human Genetics, Center for Molecular Medicine, Jichi Medical University, 3311-1 Yakushiji, Shimotsuke, Tochigi 329-0498 Japan; 2 Laboratory of Evolutionary Anthropology, Department of Integrated Biosciences, Graduate School of Frontier Sciences, The University of Tokyo, 5-1-5 Kashiwanoha, Kashiwa, Chiba 277-8562 Japan; 3 Division of Structural Biochemistry, Department of Biochemistry, School of Medicine, Jichi Medical University, 3311-1 Yakushiji, Shimotsuke, Tochigi 329-0498 Japan

**Keywords:** Biochemistry, Molecular medicine

## Abstract

*Ildr2* was initially identified as a genetic modifier of diabetes susceptibility in B6.DBA *Lep*^*ob*^ congenic mice, and was associated with decreased β-cell replication rates, reduced β-cell mass, and persistent mild hypoinsulinemic hyperglycemia. However, the molecular mechanisms of how the ILDR2 protein is involved in these effects are largely unknown. We sought to identify ILDR2-interacting proteins to further elucidate the molecular mechanisms underpinning ILDR2 function in pancreatic β-cells. Using TAP tag technology, we purified proteins interacting with ILDR2 in the pancreatic β-cell line MIN6, and identified the endoplasmic reticulum resident chaperones, GRP78 and PDIA1, as novel proteins interacting with ILDR2. We demonstrated that GRP78 interacted with ILDR2 and was possibly involved in ILDR2 stabilization by inhibiting ubiquitin–proteasome degradation. Additionally, adenoviral ILDR2 knockdown led to reduced glucose-responsive insulin secretion in MIN6 β-cells, suggesting ILDR2 may be implicated in a new pathway in hypoinsulinemic hyperglycemia. These data provide evidence for a novel association between GRP78 and ILDR2, and suggest GPR78-ILDR2 may a novel target for diabetic therapeutic modulation in decreased insulin secretion.

## Introduction

Immunoglobulin-like domain-containing receptor 2 (*Ildr2*) is a diabetes susceptibility gene encoding a transmembrane protein localized to the endoplasmic reticulum (ER) membrane ^1^. The gene transcript is ubiquitously expressed, especially at tissue associated with diabetes development, including the hypothalamus, liver, and pancreatic islets ^2^. We previously reported that adenovirus-mediated *Ildr2* transduction in the liver of *ob/ob* obese model mice mitigated the effects of hepatic steatosis^1^. Our data suggested that the ILDR2 protein played a role in the hepatic clearance of lipoproteins, and was involved in hepatic lipid homeostasis.

ILDR2 is a member of anglin protein family, and is required for three-cell-contact where the three cell horns of epithelial cell sheets meet^3^. ILDR2 is also reported to function as a B7-like protein family member expressed on immune cells and inflamed tissue, with T-cell inhibitory activity, and involved in the development of immune-related diseases such as autoimmunity and cancer^4^. In an animal model of autoimmune disease, treatment with ILDR2-Fc, which fused the extracellular domain of ILDR2 with the fragment crystallizable (Fc) portion of immunoglobulin G (IgG), was improved by regulating immune homeostasis and inducing antigen-specific immune tolerance^5^.

*Ildr2* was initially identified as a genetic modifier of diabetic susceptibility in B6.DBA *Lep*^*ob*^ congenic mice, and was associated with decreased β-cell replication rates, accompanied by reduced β-cell mass, and persistent mild hypoinsulinemic hyperglycemia^2^. However, the molecular mechanisms underpinning ILDR2 involvement in these roles remain unclear.

In this study, we aimed to identify glucose-regulated protein 78 (GRP78) as an ILDR2 interacting protein and demonstrated that GRP78 could contribute to ILDR2 stabilization and increased GRP78 levels during ER stress, thereby preventing ILDR2 decline. The identification of an association between decreased ILDR2 expression in β-cells and decreased insulin secretion may potentially represent a component of a new pathway in hypoinsulinemic hyperglycemia and provide a new mechanism for decreased insulin secretion.

## Results

### ILDR2 forms a complex with the ER chaperone proteins, GRP78 and protein-disulfide isomerase, PDIA1

**Figure 1:**
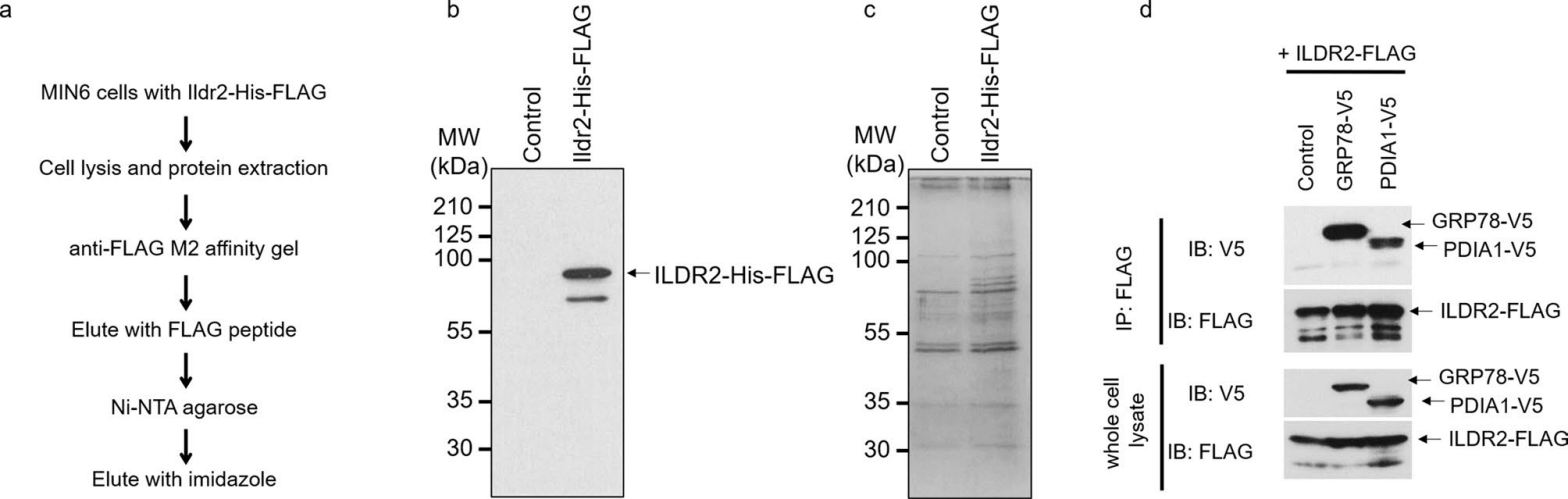
ILDR2 interacts with GRP78 and PDIA1. (**a**) Purification strategy for ILDR2-containing protein complexes using the tandem affinity purification technique. (**b**) ILDR2-His-FLAG was immunoblotted using an anti-FLAG antibody. (**c**) ILDR2-containing protein complexes were purified from MIN6 cells infected with His-FLAG-tagged ILDR2 adenovirus. Protein complexes were separated by SDS-PAGE and detected by silver staining. ILDR2-interacting proteins were identified by MALDI-TOF mass spectrometry (Supplementary Table 1). (**d**) HEK293T cells were transiently co-transfected with His-FLAG-tagged ILDR2, and either V5-tagged GRP78 or V5-tagged PDIA1. After co-transfection, whole cell lysates were prepared and immunoprecipitated with an anti-FLAG antibody, followed by immunoblotting with anti-FLAG or anti-V5 antibodies. *n* = 3 per group.

To identify proteins interacting with ILDR2 in pancreatic MIN6 β-cells, we performed tandem affinity purification (TAP). We infected cells with a His-FLAG-tagged ILDR2 adenovirus and purified ILDR2-containing protein complexes using FLAG antibody affinity, and nickel-agarose chromatography (Fig. 1a). The purified His-FLAG-tagged ILDR2 was immunoblotted with an anti-FLAG antibody (Fig. 1b). ILDR2-interacting proteins were separated using SDS-PAGE, and visualized by silver staining (Fig. 1c). These ILDR2-interacting proteins were identified by MALDI-TOF mass spectrometry (Supplementary Table 1).

ILDR2 interacted with the ER chaperone proteins, GRP78, and the protein-disulfide isomerase, PDIA1. Of all the proteins that interacted with ILDR2, we focused on these two because GRP78, PDIA1, and ILDR2 localized to the ER ^1^. We next performed immunoprecipitation studies to ascertain whether ILDR2 physically bound to GRP78 and PDIA1 in the intracellular region. We transiently co-transfected HEK293T cells with FLAG-tagged ILDR2 and either V5-tagged GRP78 or V5-tagged PDIA1. After co-transfection, whole cell lysates were prepared and immunoprecipitated with appropriate anti-FLAG antibodies. We observed that ILDR2 interacted with GRP78 and PDIA1 (Fig. 1d).

### ILDR2 expression is affected by GRP78 and PDI inhibitors

**Figure 2:**
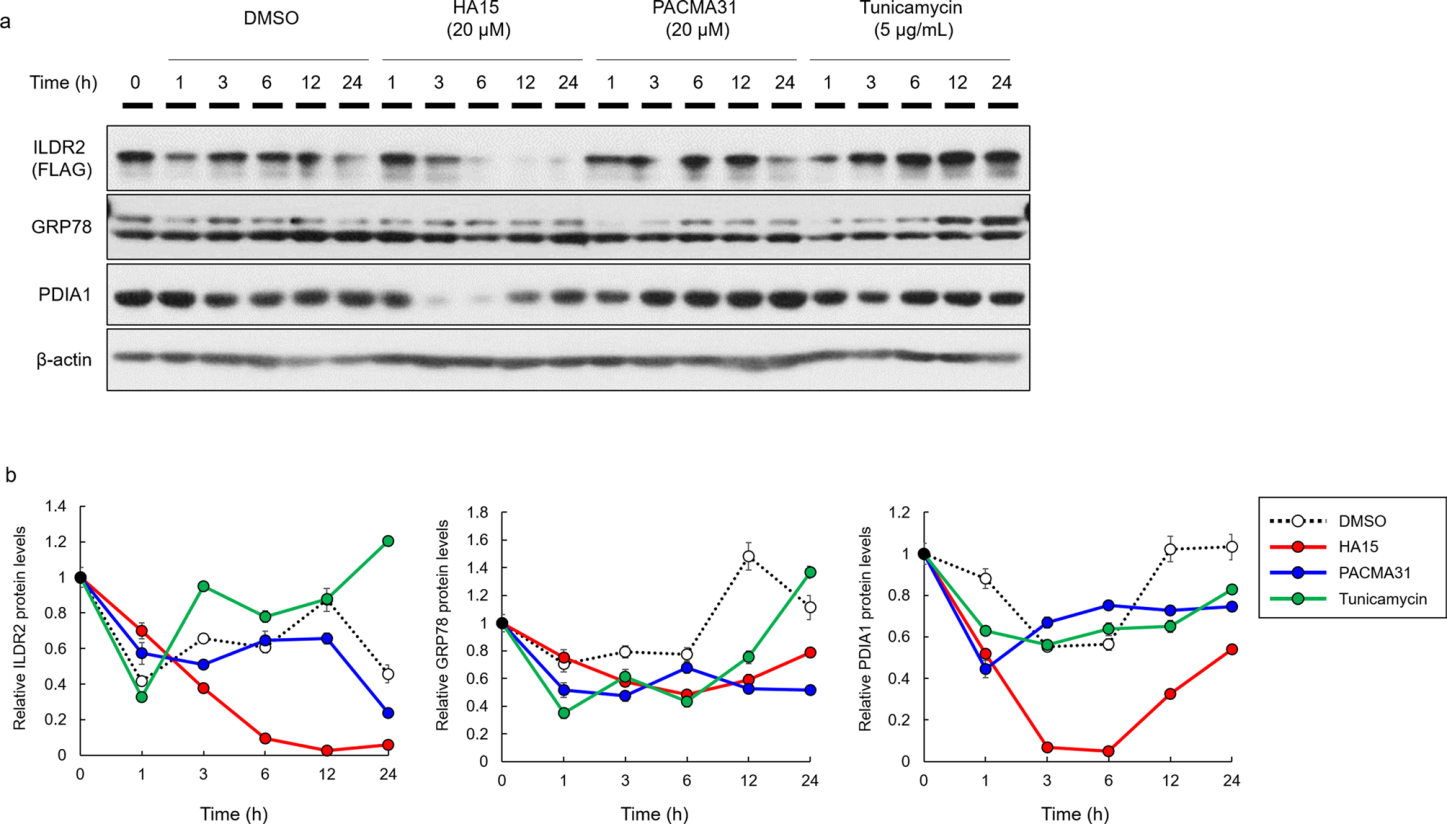
The effects on ILDR2 expression using GRP78 and PDIA1 inhibitors. (**a**) MIN6 cells were infected with FLAG-tagged ILDR2 adenovirus. After 48 h infection, cells were incubated with or without HA15, PACMA31, and tunicamycin at each time point, and whole cell lysates were prepared and subjected to western blotting using anti-FLAG, anti-GRP78, anti-PDIA1, and anti-β-actin antibodies. *n* = 3 per group. (**b**) Quantification of relative protein levels for ILDR2, GRP78, and PDIA1 by western blot. The data were normalized with β-actin levels. *n* = 3 per group. Statistical analysis data were presented in Supplementary Table 2.

GRP78 belongs to the heat shock protein 70 (HSP70) family, which corrects protein folding and prevents the transport of misfolded proteins^6,7^. PDIA1 is a member of the PDI family, which catalyzes both disulfide oxidation and disulfide isomerization to ensure that protein structures are correctly folded^8,9, 10^. Since both GRP78 and PDIA1 were important for correct protein folding, we hypothesized they could be involved in ILDR2 folding and stability. To test this hypothesis, we examined ILDR2 protein levels in MIN6 cells using GRP78 and PDI inhibitors. We infected MIN6 cells with FLAG-tagged ILDR2 adenovirus. After 48 h infection, we incubated cells with DMSO, HA15 (GRP78 inhibitor), PACMA31 (PDI inhibitor), and tunicamycin (ER stress inducer) at different time point, prepared whole cell lysates and performed western blotting using anti-FLAG, anti-GRP78, anti-PDIA1, and anti-β-actin antibodies. ILDR2 expression was decreased by the GRP78 inhibitor, HA15 in a time-dependent manner (Fig. 2). The PDI inhibitor, PACMA31 also decreased ILDR2 expression at 24 h. The ER stress inducer, tunicamycin, increased ILDR2 expression in a time-dependent manner. GRP78 expression was unchanged by GRP78 and PDI inhibitors, but was increased by tunicamycin treatment. PDIA1 expression was reduced by the GRP78 inhibitor, but was unchanged by PACMA31 and tunicamycin treatment (Fig. 2).

### Changes in ILDR2 expression affects PERK activation during ER stress

**Figure 3:**
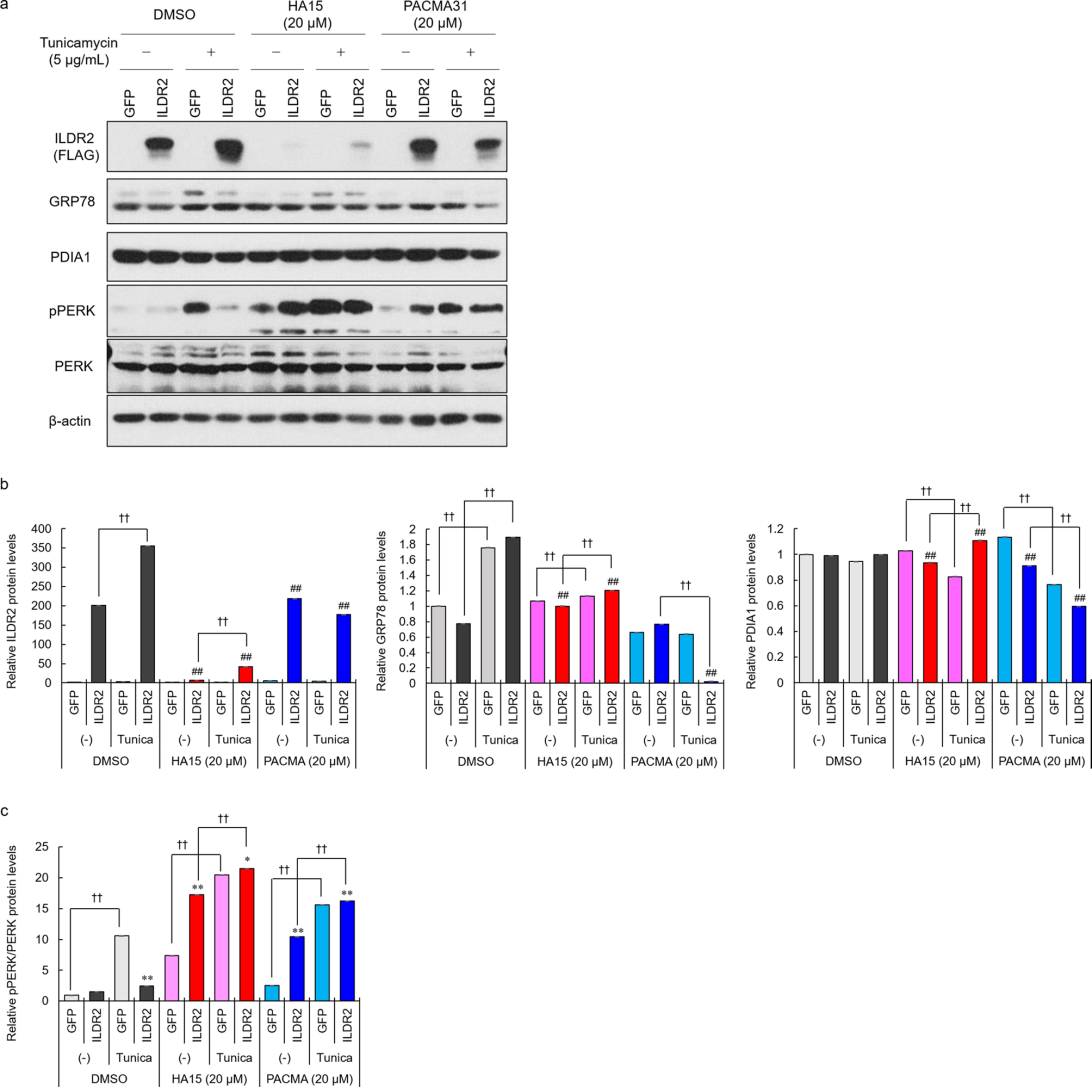
Changes in ILDR2 expression levels affect PERK activation under ER stress. (**a**) MIN6 cells were infected with either GFP or FLAG-tagged ILDR2 adenovirus. After 48 h infection, cells were incubated with or without HA15, PACMA31 and tunicamycin for 6 h, and whole cell lysates were prepared and subjected to western blotting using anti-FLAG, anti-GRP78, anti-PDIA1, anti-pPERK, anti-PERK, and anti-β-actin antibodies. *n* = 3 per group. (**b**) Quantification of relative protein levels for ILDR2, GRP78, and PDIA1 by western blotting. The data were normalized with β-actin levels. *n* = 3 per group, ^†^*p* < 0.05, ^††^*p* < 0.01 vs. Tunicamycin (-); ^#^*p* < 0.05, ^##^*p* < 0.01 vs. DMSO (**c**) Relative pPERK expression. Data were normalized to total PERK levels. *n* = 3 per group, *p < 0.05, **p < 0.01 vs. GFP; ^†^*p* < 0.05, ^††^*p* < 0.01 vs. Tunicamycin (-). Statistical analysis data were presented in Supplementary Table 3.

GPR78 inhibition by HA15 decreased ILDR2 expression, while an increase in GPR78 levels by tunicamycin increased ILDR2 expression in MIN6 cells expressing ILDR2 (Fig. 2). These data suggested that intracellular GRP78 levels and GRP78-ILDR2 interactions might be important in regulating ILDR2 levels in MIN6 cells. Therefore, we examined how increased GRP78 expression via tunicamycin treatment was affected by GRP78 and PDI inhibitors, in terms of ILDR2 expression. MIN6 cells were infected with either GFP or FLAG-tagged ILDR2 adenovirus vectors. After 48 h infection, we incubated cells with DMSO, HA15, PACMA31, with/without tunicamycin for 6 h. After this, whole cell lysates were prepared and subjected to western blotting. ILDR2 expression levels were increased by tunicamycin when compared to without tunicamycin treatment in DMSO and HA15 groups (Fig. 3). HA15 reduced ILDR2 expression levels with and without tunicamycin, but ILDR2 expression was slightly higher in the tunicamycin treatment group (Fig. 3a and b). In both GFP- and ILDR2-expressing cells, GRP78 expression was increased by DMSO and HA15 groups with tunicamycin treatment but not in the PACMA31 group. PDIA1 expression levels were unchanged in the DMSO group. HA15 with tunicamycin treatment decreased PDIA1 expression levels in GFP-expressing cells, but increased in ILDR2-expressing cells. In the PACMA31 group, PDIA1 expression levels were decreased by tunicamycin treatment (Fig. 3a and b). In our previous study, we showed that differences in ILDR2 expression in the liver affected ER stress marker expression^1^. Therefore, we examined how the function of GRP78 under ER stress affected ILDR2 levels and PERK phospholylation. In the DMSO group treated with tunicamycin, PERK activation was increased in control GFP cells, however this activation was suppressed in ILDR2-expressing cells. Equally, GRP78 inhibition in ILDR2-expressing cells in the HA15 group increased PERK activity, along with a reduction of ILDR2 expression when compared to GFP cells, and this activity was further increased by tunicamycin treatment. A similar PERK activation trend was observed in PACMA31 treated cells, and HA15 treated cells (Fig. 3a and c).

### GRP78 detachment from ILDR2 affects ILDR2 ubiquitination

**Figure 4:**
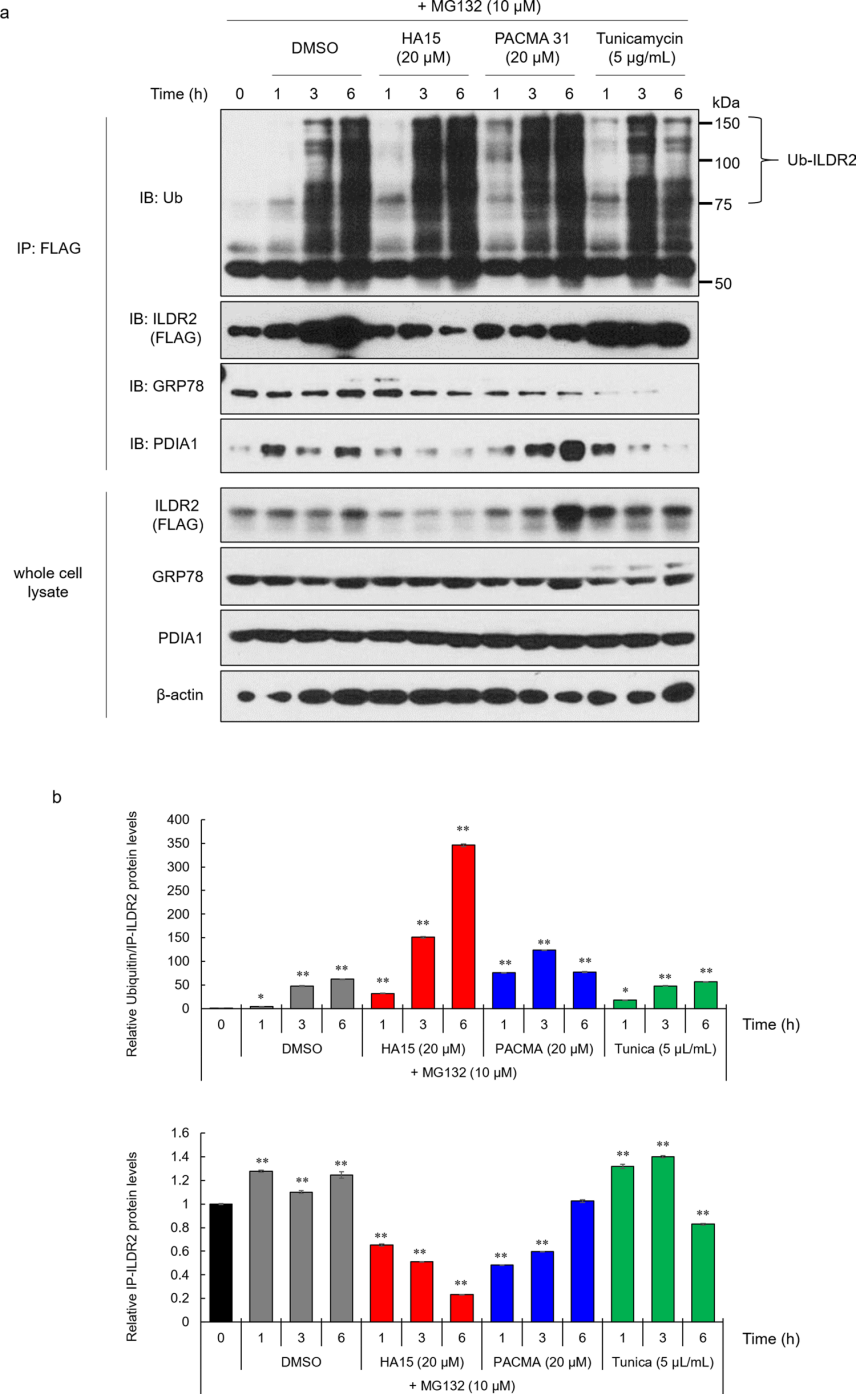
The dissociation of GRP78 from ILDR2 affects ILDR2 ubiquitination. (**a**) MIN6 cells were infected with FLAG-tagged ILDR2 adenovirus. After 48 h infection, cells were incubated with or without HA15, PACMA31, tunicamycin, and MG132 for each time point, and whole cell lysates were prepared and immunoprecipitated with an anti-FLAG antibody, followed by immunoblotting with anti-ubiquitin, anti-FLAG, anti-GRP78, or anti-PDIA1 antibodies. Western blotting of whole cell lysates was conducted using anti-FLAG, anti-GRP78, anti-PDIA1, and anti-β-actin antibodies. *n* = 3 per group. (**b**) Quantification of relative protein levels of ILDR2 and ILDR2 ubiquitination. The data were normalized to ILDR2 levels. *n* = 3 per group, *p < 0.05, ** *p* < 0.01 vs. 0 h Statistical analysis data were presented in Supplementary Table 4.

Reduced ILDR2 expression induced by the GRP78 inhibitor was believed to have arisen from GRP78-ILDR2 complex inhibition. To confirm whether the reduced interaction of GRP78 to ILDR2 was involved in potential ILDR2 degradation, we examined ILDR2 ubiquitination using the proteasome inhibitor MG132. MIN6 cells were infected with FLAG-tagged ILDR2 adenovirus. After 48 h infection, cells were incubated with DMSO, HA15, PACMA31, tunicamycin, and MG132 at each time point. To analyze ubiquitinated ILDR2, we performed immunoprecipitation with anti-FLAG antibodies, followed by western blotting with an anti-ubiquitin antibody (Fig. 4a). ILDR2 ubiquitination levels increased in a time-dependent manner in DMSO control cells. In HA15-treated cells, ILDR2 expressions in both FLAG immunoprecipitates and whole cell lysates were reduced and ILDR2 ubiquitination levels were markedly increased at 6 h. In PACMA31-treated cells, ILDR2 expression in FLAG immunoprecipitates was decreased from 1 to 3 h, and ILDR2 ubiquitination levels gradually increased from 1 h. In tunicamycin-treated cells, ILDR2 expressions in both FLAG immunoprecipitates and whole cell lysates were increased from 1 to 3 h, but FLAG immunoprecipitates were decreased at 6 h, and ILDR2 ubiquitination levels gradually increased from 1 to 6 h. GRP78 bound to ILDR2 was decreased in HA15, PACMA31, and tunicamycin-treated cells when compared with DMSO control cells. ILDR2-bound PDIA1 decreased in a time-dependent manner in HA15 and tunicamycin cells when compared to DMSO treated cells, and gradually increased in PACMA31 cells (Fig. 4a and b). These data indicated that HA15 interfered with ILDR2 stabilization possibly by inhibiting GRP78, and further promoted ILDR2 ubiquitination, suggesting inhibition of the GRP78-ILDR2 interaction was involved in ILDR2 degradation at the proteasome.

### ILDR2-knockdown reduces GSIS in MIN6 cells, and activates PERK

**Figure 5:**
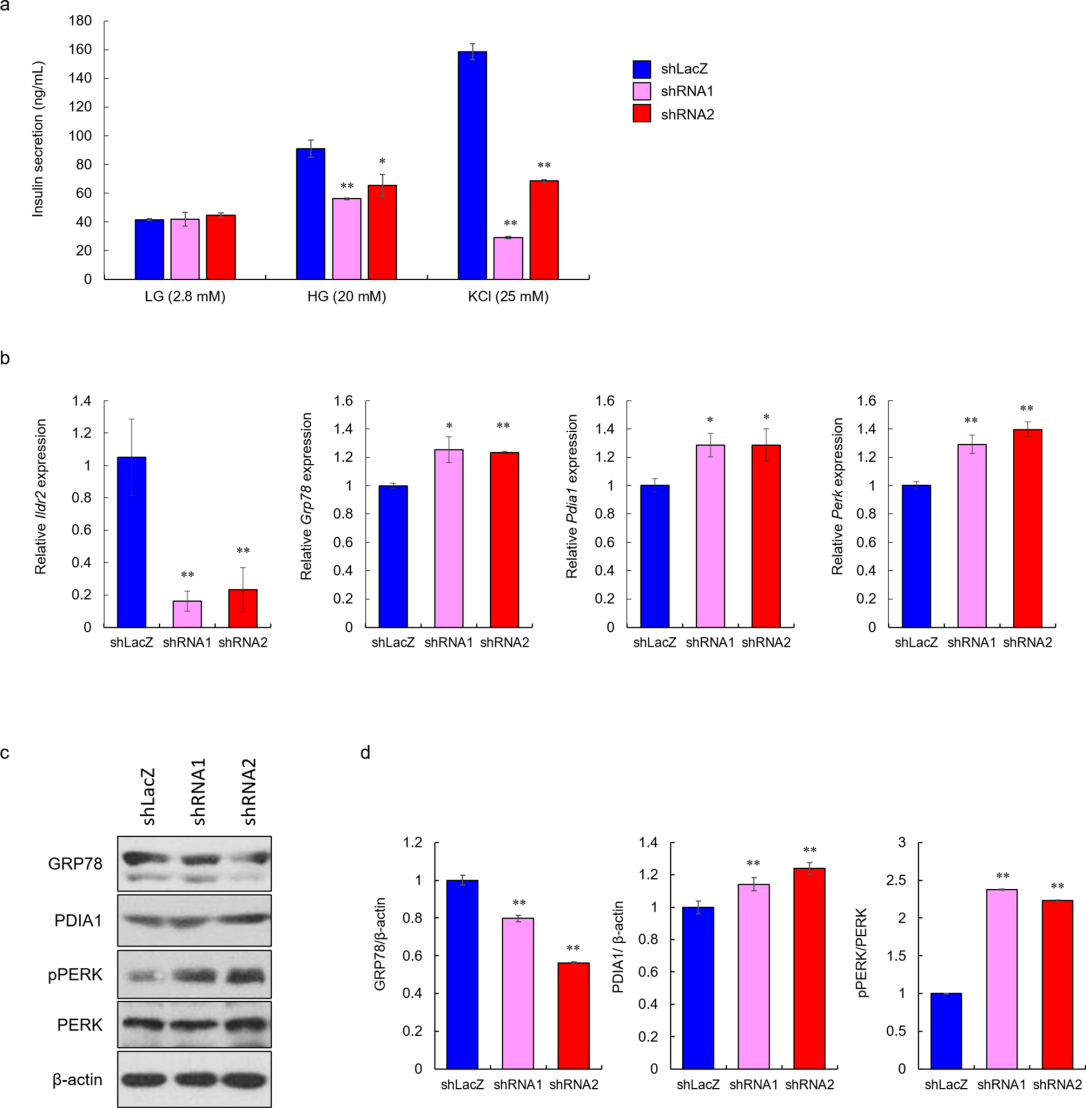
ILDR2 knockdown reduces glucose-stimulated insulin secretion (GSIS) in MIN6 cells, and activates PERK. MIN6 cells were infected with either shLacZ or *Ildr2*-shRNA (shRNA1 and shRNA2) adenovirus. (**a**) After 48 h infection, GSIS was performed. *n* = 4 per group, *p < 0.05, **p < 0.01 vs. shLacZ. (**b** and **c**) After 48 h infection, mRNA and proteins were extracted and subjected to qPCR and western blotting, respectively. *n* = 3 per group, *p < 0.05, **p < 0.01 vs. shLacZ. (**d**) Quantification of relative protein levels of GRP78, PDIA1, and phospho-PERK as by western blotting. Data were normalized with either β-actin or total PERK levels.

To investigate whether insulin secretion was affected by reduced ILDR2 expression in MIN6 cells, we performed GSIS on ILDR2-knockdown in MIN6 cells. MIN6 cells were infected with either shLacZ or *Ildr2*-shRNA (shRNA1 and shRNA2) adenovirus. At 48 h infection, GSIS analyses indicated that insulin secretion was reduced in ILDR2-knockdown cells at both high glucose and KCl stimulation levels (Fig. 5a). These data demonstrated reduced insulin secretion, suggesting that intracellular insulin levels were reduced in ILDR2-knockdown cells. Next, to examine gene and protein expressions in *Ildr2*-knockdown MIN6 cells, we performed qPCR and western blot analysis. *Ildr2* gene expression in *Ildr2*-knockdown MIN6 cells was reduced by 80% for both shRNA1 and shRNA2 against *Ildr2*, while *Grp78*, *Pdia1,* and *Perk* levels were significantly increased (Fig. 5b). Inconsistent with *Grp78* expression, GRP78 protein expressions were reduced in *Ildr2*-shRNAs compared to shLacZ control. PDIA1 expression was increased approximately 1.2-fold by *Ildr2*-knockdown. Phosphorylated-PERK (pPERK) expression was increased, and the pPERK to total PERK ratio increased more than twofold by *Ildr2*-knockdown (Fig. 5c and d). These data suggested that reduced insulin secretion in *Ildr2*-knockdown MIN6 cells was due to the induction of ER stress caused by reduced ILDR2.

## Discussion

GRP78 is the most abundant ER chaperone with the following roles; (1) maintaining the ER permeability barrier during protein translocation, (2) protein folding and assembly, and (3) targeting misfolded proteins for degradation ^12^. Of these functions, it was experimentally shown that GRP78 binding to target proteins promoted proteasome degradation^13,14^. For example, the GRP78-ApoB interaction decreased ApoB accumulation and secretion in HepG2 cells via the proteasome pathway^15^.

We examined whether modulating the GRP78-ILDR2 interaction affected ILDR2 protein stability and degradation. Thus, we first examined the effects of the GRP78 inhibitor, HA15 and tunicamycin-induced ER stress on ILDR2 expression. Upon GRP78 inhibition, ILDR2 expression was downregulated, but was upregulated under tunicamycin-induced ER chaperone stress (Fig. 2). This observation correlated with a dose-dependent increase in GRP78 in MIN6 cells by tunicamycin. In other words, GRP78 inhibition downregulated ILDR2 expression. Next, we sought to investigate whether GRP78 elevation accompanied by ER stress and GRP78 inhibition affected ILDR2 expression in overexpressing ILDR2 MIN6 cells treated with HA15 and tunicamycin. We observed that ILDR2 expression was reduced by HA15, but this ILDR2 reduction was suppressed by tunicamycin treatment when compared with cells treated with HA15 alone (Fig. 3a). Interestingly, the increase in GRP78 by tunicamycin slightly repressed the decrease in ILDR2 expression by HA15 (Fig. 3a and b). This suggested that increasing GRP78 expression partially blocked the decrease in ILDR2 expression.

In our previous report, we showed that differences in ILDR2 expression at the liver affected ER stress marker expression^1^. We therefore examined pPERK levels to investigate whether changes in ILDR2 protein levels affected PERK activation. From our data in Fig. 3, we observed that tunicamycin activated PERK by increasing pPERK, but ILDR2 overexpression decreased pPERK when compared to the GFP control in the DMSO group. GRP78 inhibition by HA15 led to ILDR2 depletion, which increased pPERK levels. HA15 was also shown to bind GRP78 to strongly activate the ER stress pathway^16^. Consistent with this report, PERK was activated in HA15-treated cells when compared to the DMSO control group. In the HA15 group, elevated ILDR2 expression was accompanied by increased GRP78 expression by tunicamycin, but this did not decrease PERK activation. Although GRP78 expression was increased by tunicamycin, we observed that a slight ILDR2 increase may not have led to decreased PERK activity because its function was blocked by HA15. These results suggested that GRP78 inhibition decreased ILDR2 expression, and this decrease also affected PERK activation. These data clearly supported the notion that GRP78 inhibition decreased ILDR2 expression levels, and increased the possibility that ILDR2 reduction was an important mechanism associated with ER stress.

**Figure 6:**
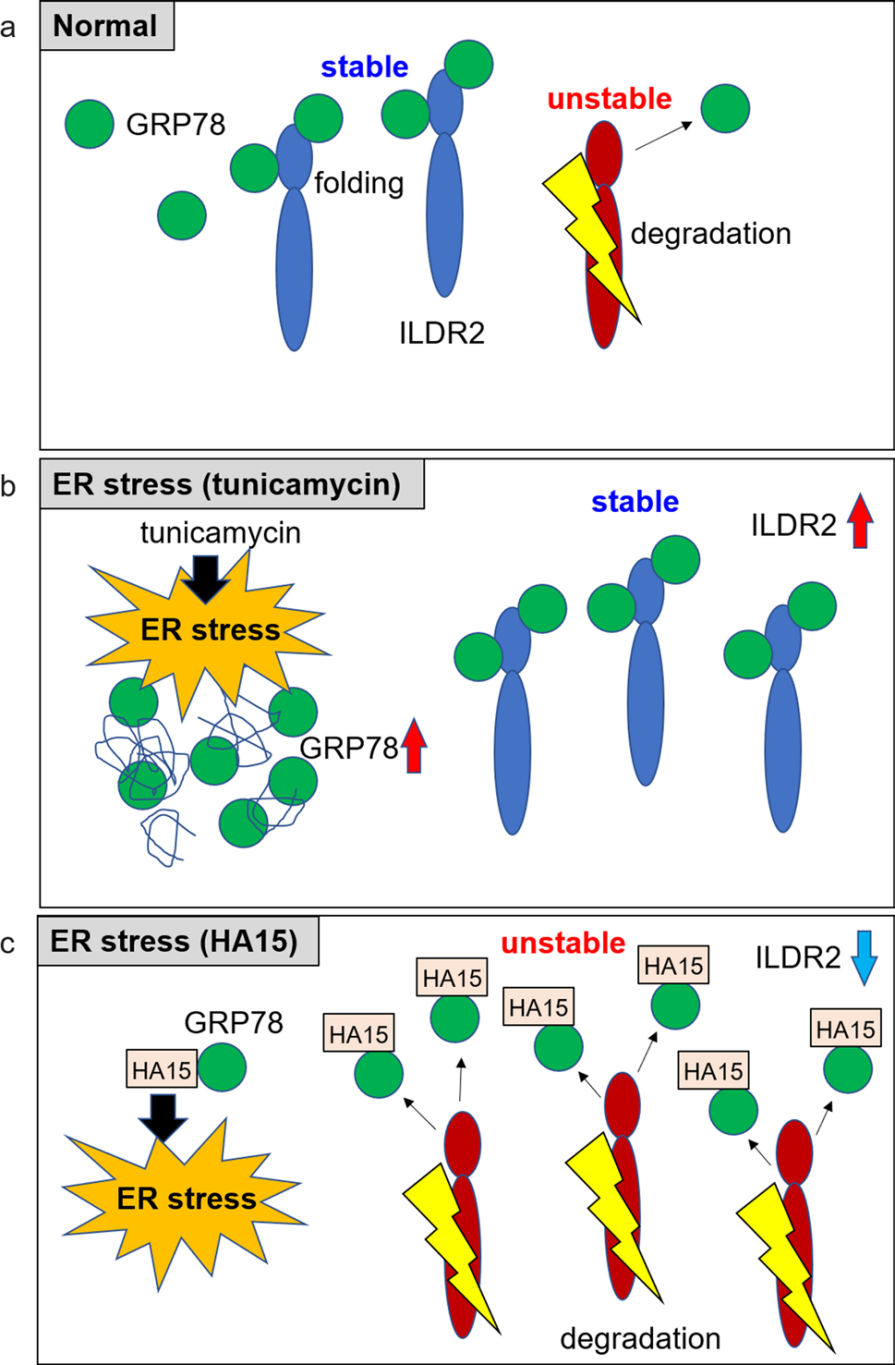
The GRP78 inhibitor, HA15 promotes ILDR2 degradation via GRP78-ILDR2 interaction. (**a**) Normal; GRP78 could be involved in the stability of ILDR2. Due to ILDR2 homeostasis in the ER, ILDR2 is released from biding to GRP78 and degraded. (**b**) Under ER stress (tunicamycin), an increase in accumulated misfolded proteins increases GRP78 levels. Consequently, GRP78-ILDR2 complex upregulation could increase ILDR2 stability. (**c**) Under ER stress (HA15), HA15 causes ER stress. HA15 binds to GRP78 and inhibits the interaction between GRP78 and ILDR2. GRP78 is released and ILDR2 becomes unstable and is degraded.

Although we indicated that GRP78 inhibition led to ILDR2 depletion, it has not been shown experimentally that inhibition of the GRP78-ILDR2 interaction could promote ILDR2 degradation at the proteasome. Therefore, we examined whether inhibition of the GRP78-ILDR2 interaction was involved in proteasome degradation of ILDR2. We examined the ubiquitination of ILDR2 using the inhibitor, MG132 and found that ubiquitinated ILDR2 was increased by HA15 treatment, indicating that GRP78 inhibition promoted ILDR2 proteasome degradation. Interestingly, the relative protein ratios of ubiquitinated ILDR2 to immunoprecipitated ILDR2 revealed markedly increased ubiquitinated ILDR2 in HA15-treated cells (Fig. 4b), suggesting GRP78 inhibition increased ILDR2 sensitivity to proteasome degradation. In addition, immunoprecipitated ILDR2 levels were increased from 1 to 3 h by tunicamycin treatment when compared with DMSO controls but were decreased at 6 h, suggesting that this was because the GRP78-ILDR2 interaction was markedly reduced at 6 h in the tunicamycin with MG132 treatment group (Fig. 4a and b). These results suggested that the GRP78-ILDR2 interaction could be involved in ILDR2 stability, and affected proteasomal degradation of ILDR2 (Fig. 6).

*Ildr2* was originally identified as a genetic modifier of diabetes susceptibility in B6.DBA *Lep*^*ob*^ congenic mice, and was associated with a reduced rate of β-cell replication, reduced β-cell mass, and persistent mild hypoinsulinemic hyperglycemia^2^. Our GSIS assays using *Ildr2*-knockdown in MIN6 cells showed that reduced *Ildr2* expression resulted in reduced glucose-responsive insulin secretion, and suggested that reduced insulin levels in these cells. Although these data were not complete evidence that directly proved β-cell mass or hypoinsulinemic hyperglycemia in B6.DBA *Lep*^*ob*^ congenic mice, they did support these findings. However, how ILDR2 was involved in these effects, and its associated molecular mechanism remains unknown. Although it is unclear why reduced ILDR2 expression levels in mouse β-cells are involved in reduced β-cell mass and insulin secretion, these data on reduced ILDR2 expression via inhibition of GRP78 and its reduction in glucose-responsive insulin secretion may help in explaining this mechanism. In the future, using β-cell specific *Ildr2*-knockout mice, we will examine how *Ildr2* reductions cause decreased β-cell replication rates, decreased β-cell mass, and persistent mild hypoinsulinemia.

In this study, PDIA1 interacted with ILDR2. However, we observed no data to explain ILDR2 protein stability, e.g., maintenance of ILDR2 protein homeostasis or degradation by the proteasome as seen for the GRP78-ILDR2 interaction. Therefore, it is currently unclear how the PDIA1-ILDR2 interaction functions in cells. However, previous reports have suggested that PDIA1 interacted with MTP and ApoB, and was associated with structural stabilization and solubilization of this complex^17,18^, therefore PDIA1 could contribute to the structural stabilization of ILDR2. Thus, the relationship between ILDR2 and PDIA1 will be comprehensively explored in the future.

In summary, we demonstrated that GRP78 interacted with ILDR2 and could be involved in stabilizing ILDR2 expression. ILDR2 degradation in MIN6 cells was inhibited by elevated GRP78 via tunicamycin supplementation, but this inhibition appeared to be incomplete, suggesting ILDR2 may be affected by GRP78 overexpression. In addition, reduced ILDR2 expression caused reduced insulin secretion from MIN6 cells. Therefore, GRP78 overexpression may also affect ILDR2 functions, including insulin secretion from MIN6 cells via interaction of GRP78 with ILDR2. The association between suppressed ILDR2 expression in β-cells and decreased insulin secretion may be an important component of a new pathway in hypoinsulinemic hyperglycemia, and thus may be a target for diabetic therapeutic modulation for decreased insulin secretion.

## Materials and methods

### Chemicals

HA15 was purchased from Sigma-Aldrich; PACMA31 and tunicamycin were purchased from Cayman Chemical; MG-132 was purchased from Chemscene.

### Cell culture

MIN6 cells (a kind gift from Dr. Rudolph Leibel, Columbia University) were maintained in high-glucose Dulbecco’s modified Eagle’s medium (DMEM) supplemented with 15% fetal bovine serum (FBS, Sigma-Aldrich F7524), 100 units penicillin/streptomycin, and 71.5 M β-mercaptoethanol at 37 °C in 5% CO_2_. HEK293T cells were maintained in high-glucose DMEM supplemented with 10% FBS and 100 units penicillin/streptomycin at 37 °C in 5% CO_2_.

### Adenoviral expression vectors

Adenoviruses were prepared using the ViraPower Adenoviral Expression System (Invitrogen), as previously described^1,19, 20,21^. PCR-amplified, C-terminal 6xHis and 3xFLAG tagged full-length *Ildr2* was subcloned into the pENTR/D-TOPO vector using the pENTR Directional TOPO Cloning Kit (Invitrogen). Inserts of pENTR-Ildr2-6xHis-3xFLAG vector were transferred into the pAd/CMV/V5-DEST vector by the Gateway system (Invitrogen).

For the shRNAs of *Ildr2* and *LacZ* were cloned into BLOCK-iT U6 entry vector (Invitrogen). The sequence of the shRNA for *Ildr2* shRNA1 was: 5′- cacc GAAGAAGGTGGCCATGCTC acgtgtgctgtccgt GAGCATGGCCACCTTCTTC -3′ and *Ildr2* shRNA2 was: cacc GCTGATTTCAAATCTTAGT gcgcttcctgtcacgc ACTAAGATTTGAAATCAGC. The inserts of pENTR shRNAs vectors were transferred into the adenovirus vector pAd/PL-DEST using the Gateway system (Invitrogen). The recombinant adenoviruses were purified by the Adenovirus Purification Miniprep Kit (Cell Biolabs).

### Tandem affinity purification and identification of ILDR2-interacting proteins

MIN6 cells infected with adenovirus vector encoding tandem affinity purification-tagged ILDR2 proteins were collected and suspended with lysis buffer (10 mm Tris–HCl (pH 8.0), 150 mM NaCl, 5 mM EDTA, 0.5% NP-40, and protease inhibitor cocktail (Roche))*.* Lysates were centrifuged at 15 000 × g at 4 °C for 20 min, and the resulting supernatants were loaded into Anti*-*FLAG M2 agarose affinity gel (Sigma-Aldrich) and rotated for 60 min at 4 °C. The resin was washed five times with wash buffer (10 mm Tris–HCl (pH 8.0), 150 mM NaCl, 5 mM EDTA, 0.2% NP-40, and protease inhibitor cocktail), and bound proteins were eluted with 150 μg/ml 3xFLAG peptide in binding buffer (50 mM NaH_2_PO_4_ and 300 mM NaCl) for Ni–NTA purification system. Eluted fractions were next loaded into Ni–NTA agarose (Qiagen) and rotated for 60 min at 4 °C. The resin was washed five times with binding buffer and then the ILDR2 protein complexes were eluted with in elution buffer (50 mM NaH_2_PO_4_, 300 mM NaCl, and 250 mm imidazole). The ILDR2 protein complexes were separated by SDS–polyacrylamide gel electrophoresis (SDS-PAGE) and transferred to polyvinylidene difluoride (PVDF) membrane. The protein bands of interest were excised from the PVDF membrane and digested with trypsin. The trypsinized peptides were then treated with 50% acetonitrile containing 0.1% trifluoroacetic acid and desalted with a Ziptip C18. The peptides were analyzed by matrix-assisted laser desorption-ionization/time of flight mass spectrometry (MALDI/TOF–MS), Autoflex (Bruker Daltonics). Detected masses and the sequences of the peptides were subjected to database searches using Mascot software (Matrix Science)^11^.

### Western blot

Whole cell lysates were separated by SDS-PAGE and transferred to PVDF membranes. Proteins were detected with FLAG (F1804, Merck), GRP78 (sc-13539, Santa Cruz Biotechnology), PDI (sc-74551, Santa Cruz Biotechnology), phospho-PERK (3179 Cell Signaling Technology), PERK (sc-13073, Santa Cruz Biotechnology), Ub (sc-8017, Santa Cruz Biotechnology), and β-actin antibody (G043, abm).

### Silver staining

The silver staining was performed using Silver Stain MS Kit (FUJIFILM Wako Pure Chemical Corporation) according to the manufacturer’s protocol.

### Glucose-stimulated insulin secretion (GSIS) assay

MIN6 cells were infected with either shLacZ or *Ildr2* shRNA (shRNA1 and shRNA2) adenovirus. After 48 h of adenovirus infection, GSIS was performed.

For GSIS, adenovirus infected MIN6 cells were pre-incubated in Krebs–Ringer bicarbonate buffer (KRBH) containing 0.5% bovine serum albumin (BSA) and 2.8 mM glucose. Then, they were exposed to 2.8 mM glucose (low glucose; LG) or 20 mM glucose (high glucose; HG) or 25 mM KCl in KRBH with 0.5% BSA for 30 min. Insulin levels were determined with the Mouse Insulin ELISA Kit (Morinaga).

### Real-Time PCR (qPCR)

Total RNAs were isolated using acid guanidinium thiocyanate–phenol reagent, as previously described^11^. cDNA was synthesized using the Verso cDNA Kit (Thermo Fisher Scientific) with random hexamer primers. qPCR assays were performed using the ViiA7 Real-Time PCR System, as previously described ^11^. The relative gene expression levels were quantified with qPCR, followed by normalization to ribosomal protein lateral stalk subunit P0 (*Rplp0*) as the internal control gene. The primers used for this analysis were listed in Supplementary Table 5.

### Statistical analysis

Statistical significance was tested using one-way ANOVA with Bonferroni test for multiple comparisons. Data were expressed as mean ± SEM. Statistical significance was set at *p* < 0.05 and indicated by *p < 0.05, **p < 0.01.

## Supplementary Information

Supplementary Information 1.

Supplementary Information 2.

Supplementary Information 3.
