# Supplementary material for: ILDR2 stabilization is regulated by its interaction with GRP78
Source: Sci Rep. 2021 Apr 16;11:8414. doi: 10.1038/s41598-021-87884-7 (PMC8052334; doi:10.1038/s41598-021-87884-7)
Supplement: Supplementary file 1 — Supplementary Information 1. [file 41598_2021_87884_MOESM1_ESM.docx]

**Supplementary Information**

**ILDR2 stabilization is regulated by its interaction with GRP78**

Kazuhisa Watanabe^1*^, Kazuhiro Nakayama^2^, Satoshi Ohta^3^, Ayumi Matsumoto^1^, Hidetoshi Tsuda^1^, and Sadahiko Iwamoto^1^

^1^ Division of Human Genetics, Center for Molecular Medicine, Jichi Medical University, 3311-1 Yakushiji, Shimotsuke, Tochigi 329-0498 Japan

^2^ Laboratory of Evolutionary Anthropology, Department of Integrated Biosciences, Graduate School of Frontier Sciences, The University of Tokyo, 5-1-5 Kashiwanoha, Kashiwa, Chiba 277-8562 Japan

^3^ Division of Structural Biochemistry, Department of Biochemistry, School of Medicine, Jichi Medical University, 3311-1 Yakushiji, Shimotsuke, Tochigi 329-0498 Japan

Corresponding author: Kazuhisa Watanabe, PhD

Division of Human Genetics,

Center for Molecular Medicine, Jichi Medical University, 3311-1 Yakushiji, Shimotsuke, Tochigi 329-0498, Japan

Phone No: +81-285-58-7341

Fax No: + 81-285-44-4902

Email Address: kwatanabe@jichi.ac.jp

**Supplementary Table 1**

Identified ILDR2-interacting proteins; GRP78 and PDIA1 (protein folding-associated proteins), PPP1R36 (protein phosphatase), NLRP10 (immune response), ARHGAP5 and TBC1D9 (GTPase activating proteins), ZFP2, ZFP37, and ZSWIM4 (zinc finger proteins), and others.

**Supplementary Table 2**

Statistical analysis data for the Figure 2b.

**Supplementary Table 3**

Statistical analysis data for the Figure 3b.

**Supplementary Table 4**

Statistical analysis data for the Figure 4b.

**Supplementary Table 5**

Forward and reverse primer sequences of target genes.

**Supplementary Figures**

**Figure S1:** Original images for western blot and silver stain.
