# Supplementary figures and images for: ILDR2 stabilization is regulated by its interaction with GRP78
Source: Sci Rep. 2021 Apr 16;11:8414. doi: 10.1038/s41598-021-87884-7 (PMC8052334; doi:10.1038/s41598-021-87884-7)

Figure 1

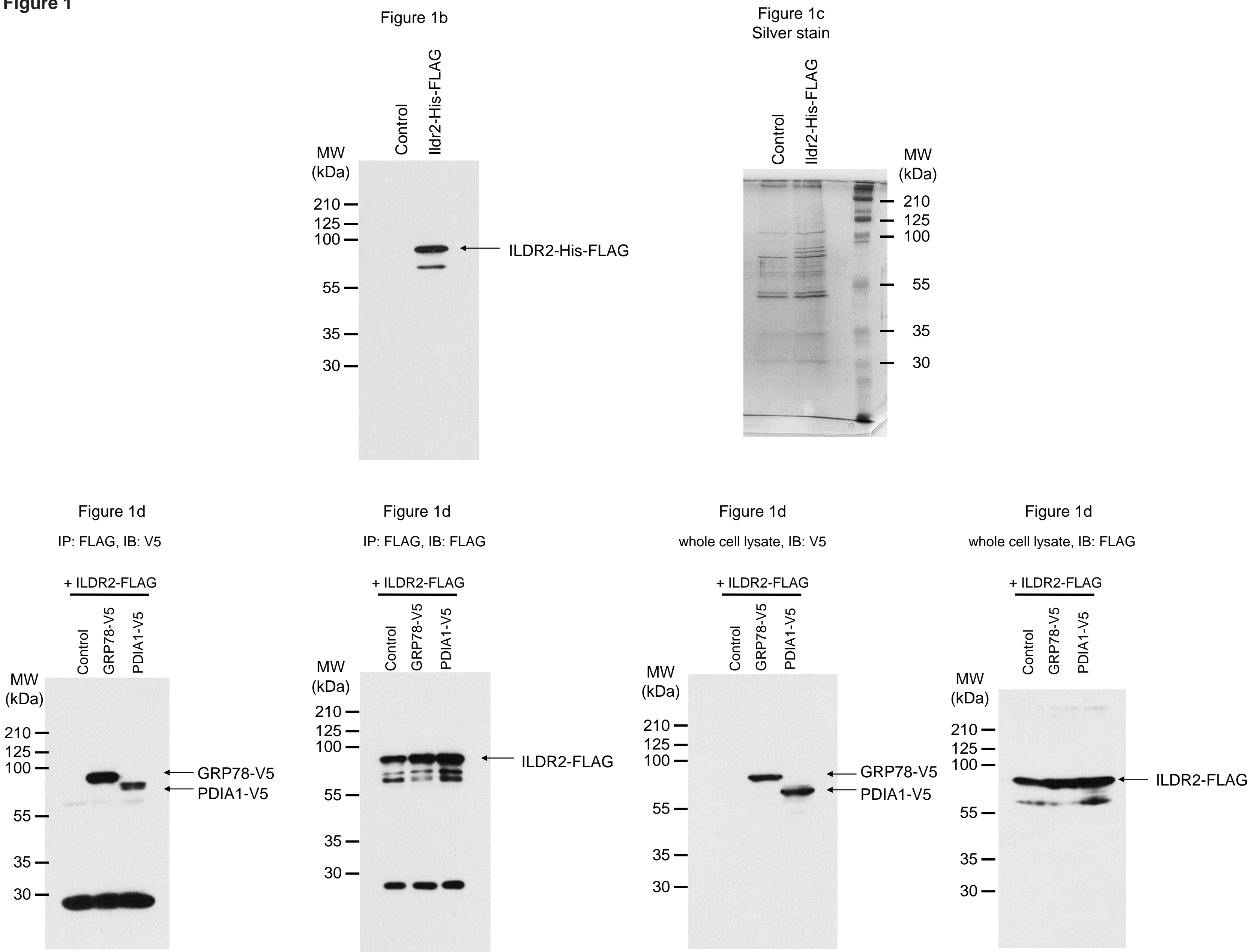

Figure 2a

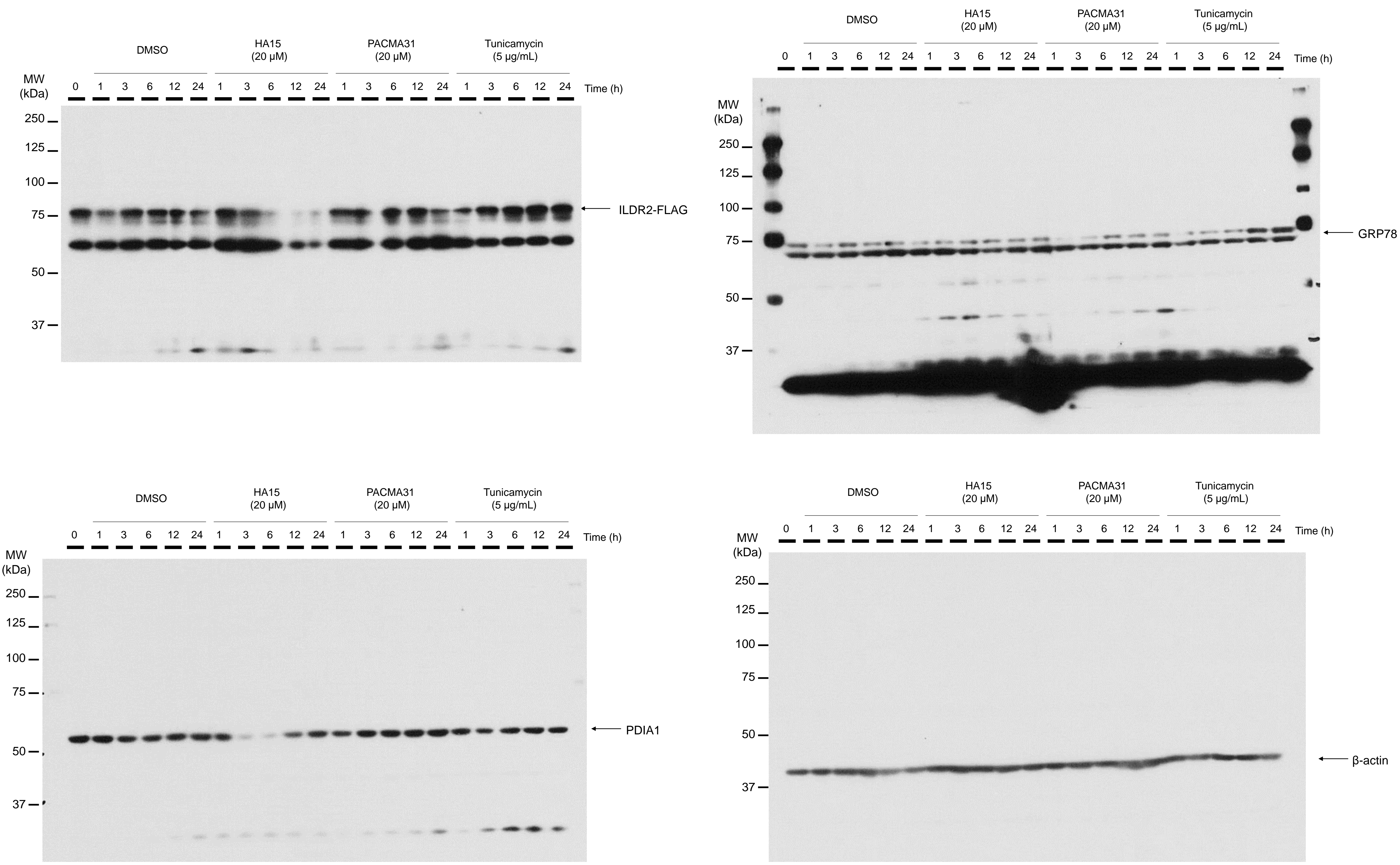

Figure 3a

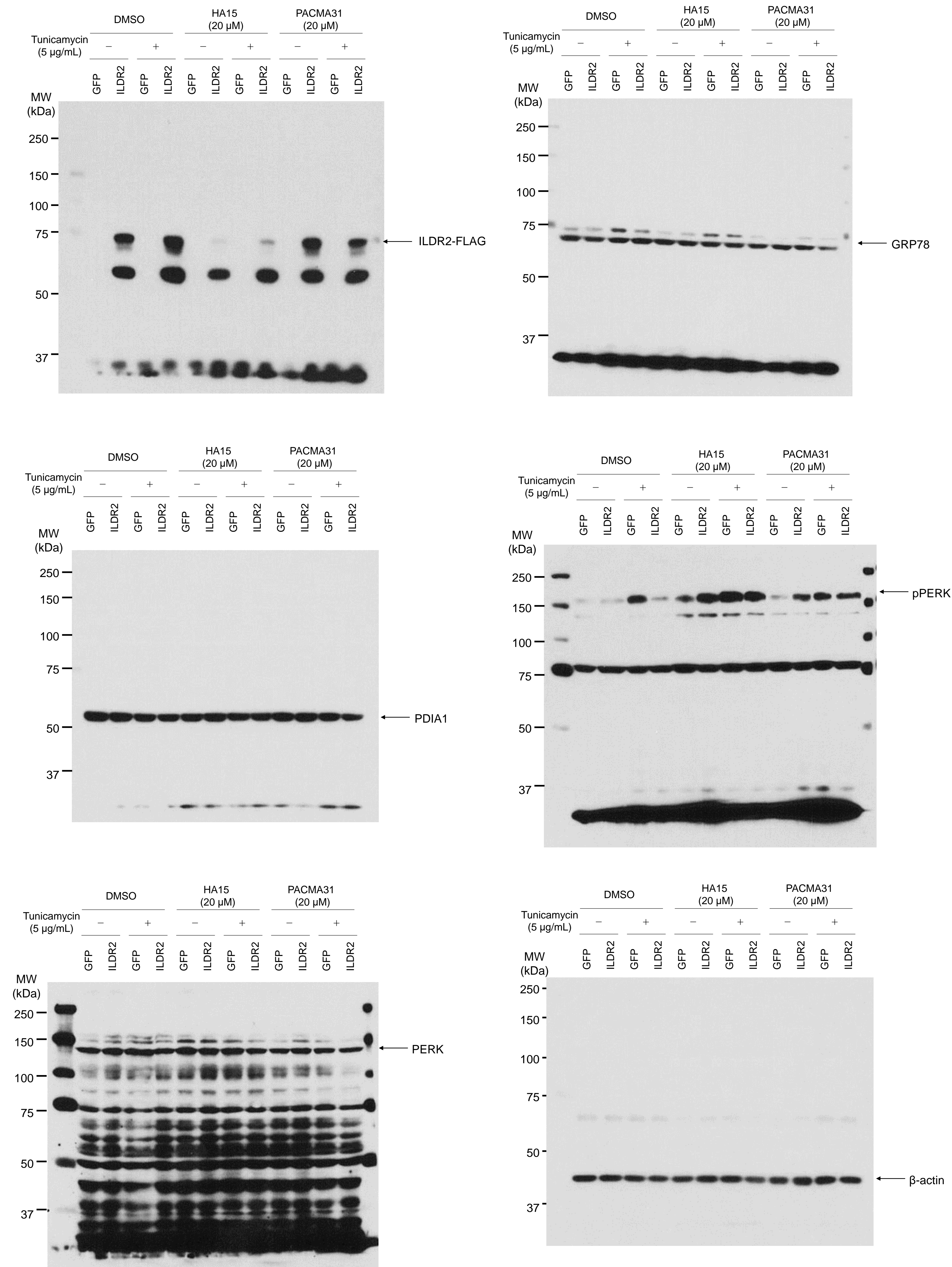

Figure 4a

IP: FLAG

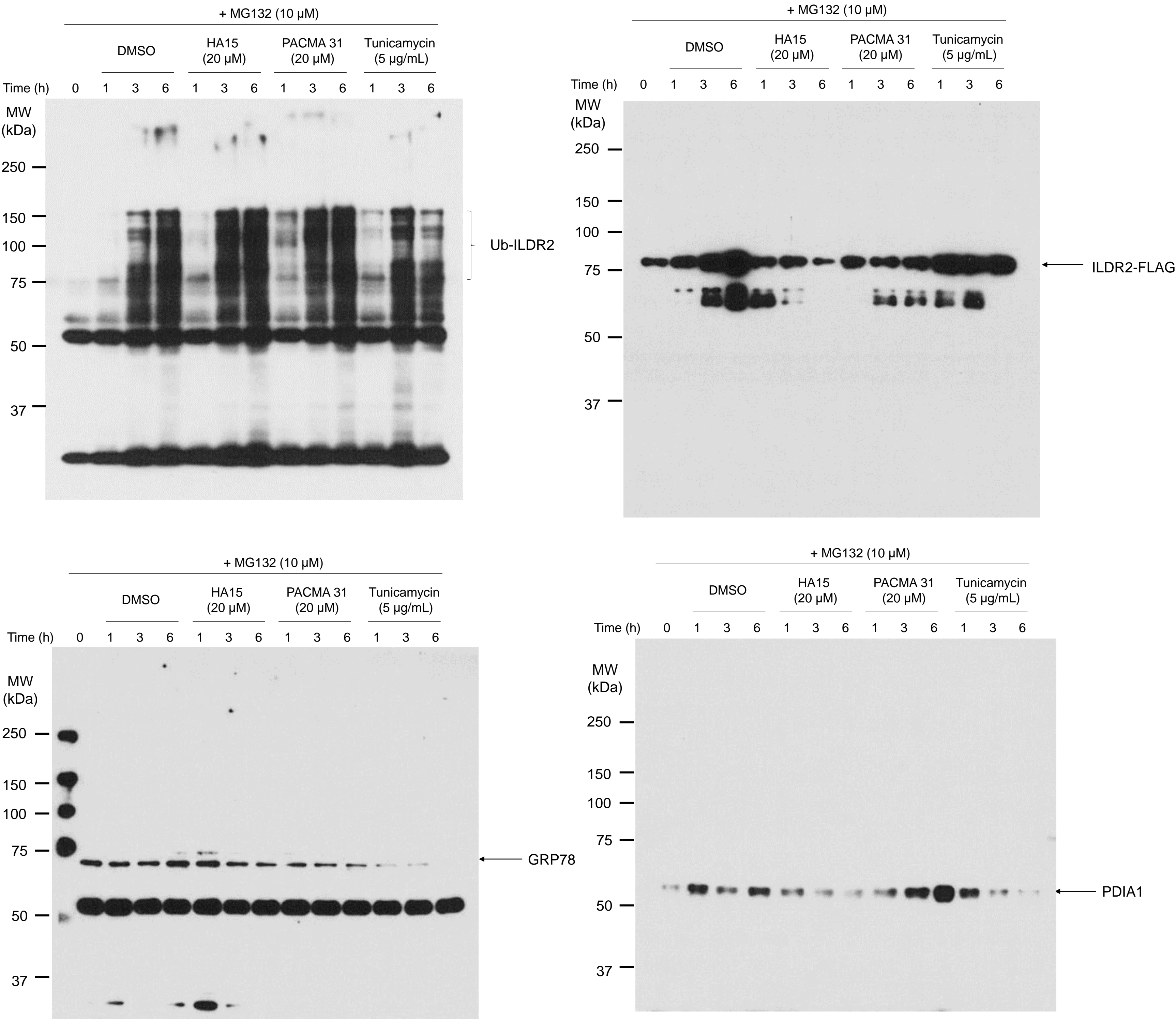

whole cell lysate

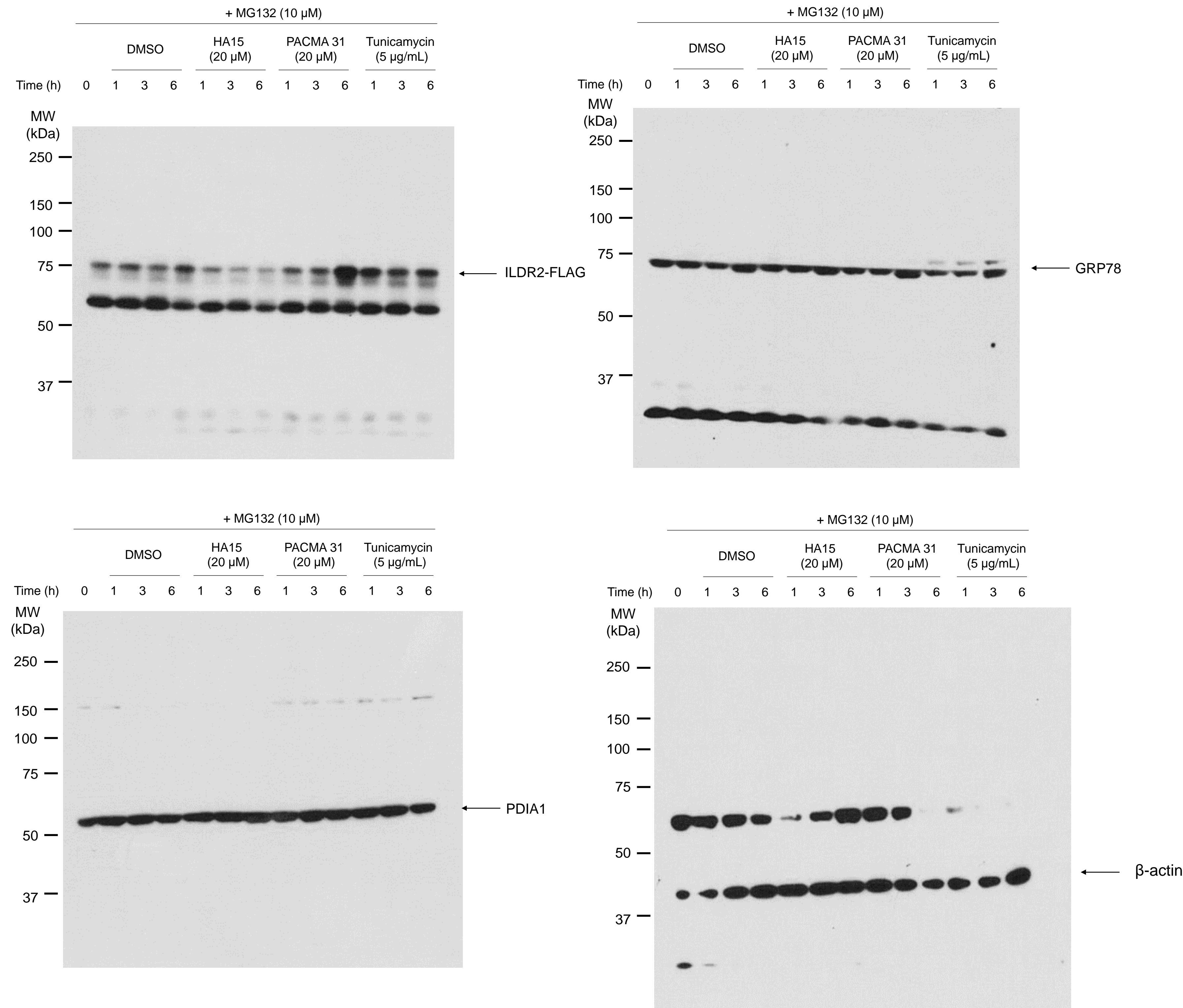

Figure 5c

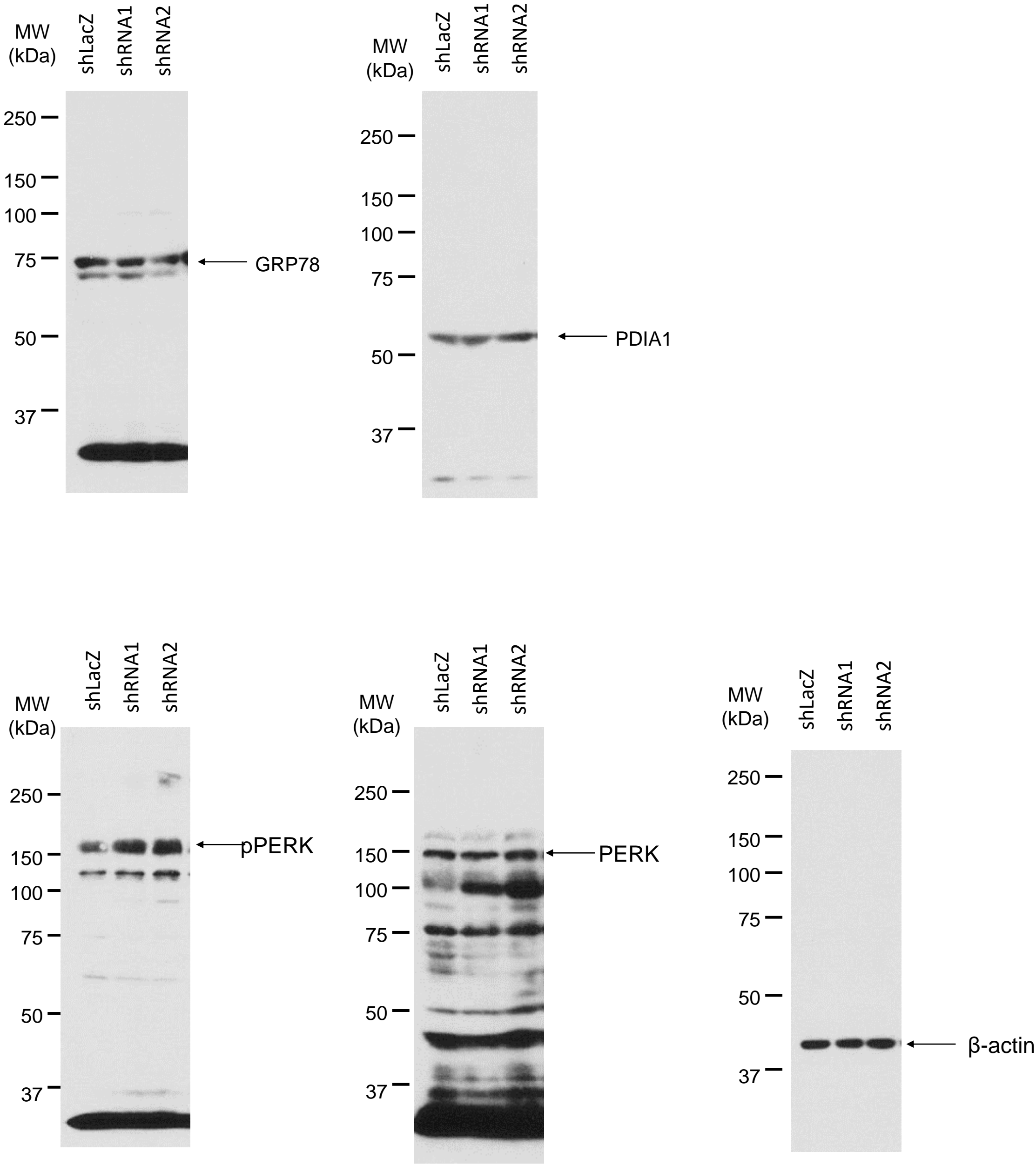

Supplement: Supplementary file 3 — Supplementary Information 3. [file 41598_2021_87884_MOESM3_ESM.pdf]
